# Interactions between self-help and hospice and palliative care – Opportunities, barriers and needs (Self-Pall): A study protocol

**DOI:** 10.1371/journal.pone.0350453

**Published:** 2026-07-09

**Authors:** Saskia Kauzner, Manuela Schneider, Julia Berendt, Christoph Ostgathe, Maria Heckel

**Affiliations:** Department of Palliative Medicine, CCC Erlangen – EMN, University Hospital Erlangen, Friedrich-Alexander-Universität Erlangen-Nürnberg (FAU), Erlangen, Germany; PLOS: Public Library of Science, UNITED KINGDOM OF GREAT BRITAIN AND NORTHERN IRELAND

## Abstract

**Introduction:**

A progressive and life-limiting disease can cause enormous psychological distress for patients and family caregivers. Health-related collective self-help could be a coping strategy by facilitating interaction with others in similar situations. Scarce literature is available on how those patients and their caregivers could benefit from self-help activities, how self-help groups deal with death and grief, and whether there are interactions between self-help and hospice and palliative care. The Self-Pall project aims at developing recommendations tailored at different actor groups to support the interactions between self-help and hospice and palliative care.

**Methods:**

We will use a qualitative, multi-method, sequential research design. The project started in 07/2025 and will end in 06/2027. Semi-structured interviews will be conducted with patients, family caregivers, and representatives of hospice and palliative care and of self-help to explore personal and professional experiences focussed on opportunities, barriers, and needs. A draft of recommendations will be derived, which will then be confirmed and expanded upon within focus groups. A representative expert panel will refine and agree upon the recommendations through an iterative, multi-level Delphi process. The engagement of a Patient and Public Involvement group will ensure the relevance of our research to the public and provide transparency.

**Discussion:**

We will explore awareness, needs, and factors that promote or hinder self-help activities from the perspective of patients, caregivers, and professionals. From a self-help perspective, we will assess how to deal with dying, death, and grief, as well as knowledge and use of hospice and palliative care services, any barriers and how to overcome them. We are the first to explore interactions between self-help and hospice and palliative care bilaterally to develop practical recommendations and key principles with significant implications for practice.

## Introduction

Being diagnosed with a serious, progressive and life-limiting disease, e.g., cancer, can be linked to enormous psychological distress for patients besides the disease-related symptom burden [1,2]. Also family caregivers – especially when caring for the ill family member at home – experience high emotional burden and this can affect health and quality of life [3,4]; they often express an unmet need for emotional and social support in their caring situation [3,5–7]. In addition to other psychosocial support services, health-related collective self-help could be supportive in the sense of a coping strategy for patients and their family caregivers as they facilitate interaction with others in a similar situation [3,8,9]. In Germany, refers to a national concept that is stipulated by law [10]. From an international perspective, we often came across concepts such as or that are similar to the German concept of self-help and refer to people with the same or a similar disease who share their lived experience [11,12]. The German professional association for self-help (Deutsche Arbeitsgemeinschaft Selbsthilfegruppe e.V.) describes the aim of collective self-help as enabling disabled and chronically ill people, as well as their caregivers, to exchange information and experiences, to promote self-determination and personal responsibility, representing their interests in public, and promoting inclusion [13]. In Germany, self-help is mainly organised through local groups, initiatives, organisations, and contact points [14]. At state or federal level, self-help support services and associations help to establish and disseminate local self-help services [15].

Positive effects of self-help for patients, e.g., with diabetes mellitus type 2, prostate cancer, multiple sclerosis, have been shown in terms of promoting empowerment [16], coping strategies, and disease-related knowledge [17]. A recent systematic review synthesised perceived benefits of participating in a peer led self-help group for cancer patients, namely informational support, shared experience, learning from others, helping others as well as cultivating humor as a coping strategy [18]. For family caregivers, participating in a support group program increased perceptions of preparedness for caregiving, competence for caregiving, and rewards of caregiving whereas there were no significant effects regarding hope, anxiety, depression symptoms and health [19].

However, limited evidence is available on how self-help could support patients with serious, progressive, and life-limiting diseases, as well as their family caregivers, at the end of life and during bereavement, and whether there are barriers. To date, self-help services for hospice and palliative care patients do barely exist in Germany and seem to be rather unknown. Furthermore, it is unclear whether patients and their caregivers recognise the need for self-help services or notice their supportive nature.

At the same time, we do not know what role hospice and palliative care play within self-help groups, especially when dealing with dying, death, and grief. Experience reports from people actively involved in German self-help services confirmed and some even reported `the tendency for self-help groups to withdraw from end-of-life care` [20]. In addition, it remains unclear how structural and political conditions influence the interaction between self-help and hospice and palliative care.

To address these research gaps, the project asks the following research questions:

1) To what extent can self-help provide support for people with serious, progressive, and life-limiting diseases and their families?
2) To what extent do the topics of dying, death, and grief, as well as hospice and palliative care, play a role in self-help?
3) To what extent do self-help services and hospice and palliative care services currently overlap?

Findings will inform recommendations and key principles to further support and strengthen the interaction between self-help and hospice and palliative care in Germany. The recommendations will be tailored to different actor groups, e.g., self-help organisations and associations, self-help activists, hospice and palliative care providers, institutions, associations, and the public to provide low-threshold guidance on different action levels.

## Methods

### Study design

**Fig 1:**
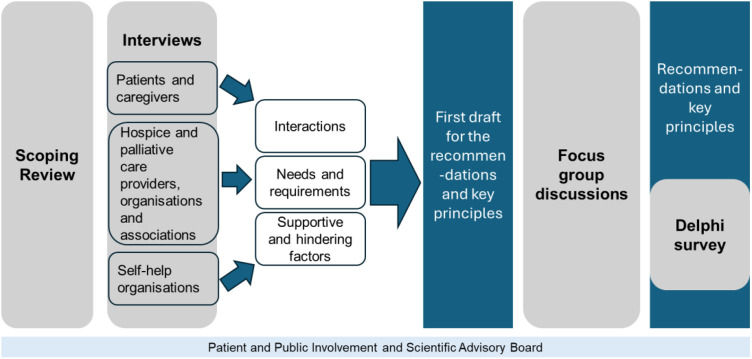
Overview of work process.

The Self-Pall project will examine the interaction between health-related collective self-help and hospice and palliative care using a qualitative, multi-method, sequential research design. The project started in 07/2025 and will end in 06/2027. We will assess personal and professional experiences with focus on opportunities, barriers, and needs by conducting semi-structured interviews with patients, informal caregivers, representatives of self-help organisations, and representatives of hospice and palliative care services and organisations. Recruitment for the interviews started in 11/2025 and will be continued during analysis of interview data until approximately 07/2026. Recommendations and key principles will be derived from the interview data. We will then use focus group discussions with representatives of each target group to confirm and adapt the first draft of the recommendations, and explore additional issues [21,22]. The recommendations will be refined and agreed upon by representative experts through an iterative, multi-level Delphi process. We expect the final recommendations to be finalized until 04/27. We will adhere to the Consolidated Criteria for Reporting Qualitative Research (COREQ) checklist [23] and the reporting standard for Conducting and Reporting of Delphi Studies (CREDES) [24]. See Fig 1 for an overview of the work process.

The engagement of a Patient and Public Involvement (PPI) group ensures that the perspectives of patients` and the community are considered, emphasise the relevance of our research to the public and provide transparency regarding the research process [25]. A scientific advisory board provides additional methodological support.

Ethics approval was granted through the Ethics Committee of the Faculty of Medicine, FAU Erlangen: 24-46-S, 20.02.2024. The study is registered at The Open Science Framework (https://doi.org/10.17605/OSF.IO/ZC23E; date of registration: 02/09/25) and the Central Study Register of University Hospital Erlangen (https://studienregister.uk-erlangen.de/details/mdpldbrrglav; date of registration: 11/09/25).

### Participants and sampling

Based on a purposeful sampling strategy, participants in interviews and focus groups will comprise different target groups in order to explore multiple perspectives and experiences: 1) Patients with serious, progressive, and life-limiting diseases and informal caregivers with personal experience of self-help; 2) Representatives of hospice and palliative care who work in patient care or who are active in related organisations or associations; 3) Representatives of self-help organisations. The Diversity Responsiveness Checklist [26] serves as an aid to encourage reflection on diversity in the team and study population at all stages of the project. A minimum age of 18 years, relevant experience in self-help and/or hospice and palliative care, and sufficient emotional, cognitive, and language skills qualifies for participation (inclusion criteria). All participants will sign an informed consent form in advance and will be informed that there is the possibility to stop the interview or leave the focus group discussion at any time.

For the Delphi survey, the expert panel will comprise confirmed or self-identified experts experienced in hospice and palliative care and/or self-help (e.g., representatives of organisations or associations), as well as individuals with roles in cities, municipalities, or health and social care institutions. We will try to ensure inter-individual variety regarding socioeconomic status, gender, and age.

Participants will be recruited via professional and personal network of the palliative care department, social media, advertisements in local press, personal contact, and snowball sampling.

### Patient and public involvement

Members of the PPI group will be recruited via personal networks and social media. Group members will act in an advisory and informative role, representing the interests of patients, caregivers and the public. Throughout the study, we plan to hold six meetings with the PPI group to discuss the development of the interview guide, the results of the interviews, and the derivation of best practice recommendations. We will adhere to the German “Checklist for Collaboration within Projects of Palliative Care Research” [27], which was developed by the German Association for Palliative Medicine.

### Data collection

#### Semi-structured interviews.

Interviews will be conducted with experts who have personal or professional experience of self-help and/or hospice and palliative care to get deeper insights in how self-help currently interacts with hospice and palliative care, supportive and hindering factors hereby, and potentially needs for future interactions. We will explore how self-help groups deal with dying, death, and grief, as well as knowledge and use of hospice and palliative care services, any fears of contact that may exist and how to overcome them. The interview guide will be developed according to Helfferich [28]. Pre-tests of the guide will be conducted with each target group to test the understandability and validity of the questions. Find more details on the interview guides for different target groups within supporting information. The interviews will take 45–60 minutes, online or in person.

#### Focus group discussions.

Participants will discuss a first draft of recommendations and key principles regarding suitable forms of self-help for patients and informal caregivers in hospice and palliative care, necessary personnel and structural conditions, and supportive factors for successful interactions between self-help and hospice and palliative care within exploratory semi-structured focus group discussions. The following topics will encourage the discussions: to what extent can care be improved through networking and possible mutual referral between hospice and palliative care, and self-help, what factors might play a key role, what limitations need to be taken into account, and at what structural levels should measures be recommended. The focus group discussions will be led by a senior researcher and will take 1.5 to 2 h. The discussion guide will be discussed and pre-tested with the PPI group. For the detailed discussion guide, see supporting information.

#### Delphi survey.

The recommendations and key principles will be refined and agreed upon in terms of relevance, completeness, and applicability by an expert panel through an iterative, multi-level Delphi technique [29]. The recommendations will be distributed to the expert panel as anonymous online survey with feedback after each round. Feedback will consist of controlled, anonymous reflection on aggregated group results concerning recommendations and key principles. Based on this feedback, participants may reflect on and, where appropriate, adjust their assessments with no obligation to change them. We expect two to three survey rounds until consensus will be reached [24]. All consented recommendations will be part of our final set of recommendations and key principles.

### Sample size calculation

We plan 15–20 interviews in total with 5–7 representatives from each out of the three target groups. The actual sample size will be guided by information power [30]. Two focus groups with 6–8 participants each will be planned (total of *n* = 16) with an inter-individual variety. The expert panel for the Delphi survey should comprise approximately 15–20 participants to ensure a representative sample of judgments while keeping the Delphi process manageable [31,32].

### Data analysis

The interviews and focus group discussions will be audio recorded with participants` permission andtranscribed verbatim. Personal data will be pseudonymised. The analysis will be conducted by two independent coders according to content-structuring qualitative content analysis following the methodology of Kuckartz [33,34] with MAXQDA version 24.11. Deductive codes will be derived based on the research questions and interview guides with openness to inductive subcodes. Deductive codes, e.g., promoting and hindering factors, suggestions for improvements, and good examples of current interactions, will be one resource for possible recommendations and will then be double coded under. Further text segments that are assessed as pointing in the direction of possible recommendations by the coders, will be coded separately under. Afterwards, all coded segments under the parental code will be clustered in terms of topic and target group. This data set will then be prioritized and drafted uniformly as recommendations within two meetings with internal researchers and PPI. Those wordings that remain unclear or controversial, will be discussed within the focus group discussions with participants of the respective target group aiming at agreeing upon the most appropriate wording. This first developed set of recommendations will be finally checked by the scientific advisory board members and PPI before we will agree to start with the Delphi process. The consensus of the Delphi survey is statistically determined by means of an approval/rejection query for each individual recommendation in the Delphi survey. A consensus is assumed if 75% of the participants in the expert panel agree [29]. For each item, the median score, standard deviation and the range will be calculated.

## Discussion

This project aims at developing recommendations and key principles regarding adequate forms of self-help for patients with serious, progressive, and life-limiting diseases and their informal caregivers. For this purpose, we will explore awareness, needs, and factors that promote or hinder self-help activities from the perspective of patients, their caregivers, and professionals in hospice and palliative care. We expect certain characteristics related to an individual`s sociocultural background, age, gender, or type of illness to influence the outcome. Our results will shed light on indications as to whether timely integration of palliative care into the course of the disease can be facilitated by self-help and to what extent collective self-help can meet the needs of patients with serious, progressive, and life-limiting diseases and informal caregivers for exchange and information, as well as for relief in the care situation. From a self-help perspective, we will explore how to deal with dying, death, and grief, as well as knowledge and use of hospice and palliative care services, and potential for barriers of using those services. With our results, we expect to further support and strengthen the interactions between self-help and hospice and palliative care in Germany to improve patient care in long-term providing recommendations tailored to different actor groups.

As a strength of our study, we will use a multi-method approach with PPI. The best practice recommendations and key principles will consider both the hospice and palliative care and the self-help perspective and will be freely accessible. Our results will be relevant and generalizable to international contexts as the concept of self-help matches international concepts of peer support. Nevertheless, culture-bound differences in peer support should be examined in more detail in future research [35]. As digitization in healthcare proceeds, self-help services are also provided more often as online format [36]. We expect our results to reveal significant insights fostering digital services as extension to traditional self-help formats.

In line with other authors, we see possible barriers for our research. Self-help is not a universal concept or programme and therefore self-help groups and activities may vary, which could affect the interpretability of our results [37]. We will ask for an individual definition of self-help in our interviews to consider this during data analysis and interpretation. In their review, Hitch et al. pointed out that possible unintended negative effects may occur in attending self-help activities, that self-help groups are not equally suitable for all individuals, and that certain intrapersonal and extra-personal conditions must be in place for them to be successful [38]. In a German study, patients who did not participate in self-help groups stated fears of negative effects, such as feeling uncomfortable in a group situation and worrying about being confronted with the suffering of others [37]. When interviewing patients and their caregivers, we will be aware of the potential for negative experiences with self-help and we will be mindful of the emotional state of patients and their caregivers when discussing personal illness or bereavement. For this reason, we will make it clear that they can stop the interview at any time and we will provide additional support if required.

There could be difficulties recruiting participants for the interviews, focus group discussions and Delphi survey. To collect data on nationwide structures and interactions, we will reach out to people across the country through our connections with German professional associations and organisations. However, we expect the majority of participants to be local, but including professional literature would cover interactions between self-help and hospice and palliative care nationwide.

## Conclusions

This study is to the best of our knowledge the first to explore interactions and gaps between self-help and hospice and palliative care bilaterally and – based on those findings – to develop practical recommendations and key principles with significant implications for practice and patient care. In doing so, we emphasise the potential of collaboration between the two healthcare actors for patients with serious, progressive, life-limiting diseases and their caregivers that has been overlooked so far. Furthermore, we hope to raise awareness of self-help and hospice and palliative care services, and to reduce the fear of seeking for help and support among those who are affected by personal illness, a care situation, or bereavement.

## Supporting information

S1 File Interview guide hospice and palliative care. Association level.
(PDF)

S2 File Interview guide hospice and palliative care. Care level.
(PDF)

S3 File Interview guide patients and relatives. (PDF)

S4 File Interview guide self-help. (PDF)

S5 File Discussion guide focus groups. (PDF)
