# Supplementary material for: Interactions between self-help and hospice and palliative care – Opportunities, barriers and needs (Self-Pall): A study protocol
Source: PLoS One. 2026 Jul 9;21(7):e0350453. doi: 10.1371/journal.pone.0350453 (PMC13349143; doi:10.1371/journal.pone.0350453)
Supplement: S1 File — Association level. (PDF) [file pone.0350453.s001.pdf]

Self-Pall: Interview guide for representatives of Hospice and Palliative care (association level)

We are delighted to have you as our interview partner. My name is xx, and I will be conducting the interview with you today. The interview will take approximately 45 to 60 minutes.

We would like to talk to you about your experience working in hospice and palliative care with self-help groups (e.g., groups, organizations, associations). Through your work at the association level, you have certainly gained insight into the overarching structures and contexts. We have prepared a few questions that we would like to ask you.

Your experiences help us to better understand the cooperation between hospice and palliative care and self-help. We want to use these findings in the Self-Pall project to develop recommendations for action. These are intended to strengthen and support cooperation between the two actors.

Do you have any questions before we start? We would like to record the interview so that we can transcribe it more accurately afterwards. Are you okay with that?

Consent forms filled out?

Turn on the recording device!

Subject area	Questions	Check – was that mentioned? If not, ask for clarification
**Description of the hospice and palliative care setup**	Could you start by briefly introducing the organization you work for and describing your role/responsibilities?	• Full-time or volunteer work?
**Self-disclosure**	Following on from this, we would be interested to know what you understand self-help to mean in the context of hospice and palliative care.	

**Role of self-help and experience with self-help within hospice and palliative care**	As the conversation progresses, we would like to focus on health-related group self-help, where people with similar conditions come together and offer each other support.<br><br>Could you please describe whether and to what extent self-help plays a role in your work?<br><br>If so, could you give some specific examples?<br>If not, why not?	• Do caregivers (family doctors) or affected individuals/relatives ask questions about self-help services?<br>• Do you see a need for self-help services in hospice and palliative care? If so, which ones?<br>• Are self-help services considered and discussed as a form of support? In what form, e.g., written information, lectures, training, contact mediation?<br>• Are you aware of any suitable self-help services?
**Cooperation between self-help and hospice and palliative care at association and organizational level**	Is there already cooperation/contact with an self-help organization at the association level?	If yes:<br>• How would you describe the collaboration?<br>• What does contact look like?<br>• How did the contact come about (family doctors)?<br>• When did it start?<br>• Who is the contact person/who are the participants?<br>If not:<br>• Why not? Are there any obstacles?<br>• Are there any plans to do so in the future?
**Factors promoting and hindering cooperation**	If yes: What works well in the collaboration, what works less well?	• Why?<br>• What framework conditions are/would be helpful?<br>• What prerequisites should be in place?

	If no: Can you imagine why no collaboration has been established so far?	
**Possibilities and limitations of self-help for seriously ill people and their relatives**	In your opinion, what kind of support could self-help provide in the area of hospice and palliative care?<br><br>What do you think self-help can and cannot achieve?<br><br>Do you know of any specific examples where self-help was helpful or hindering?	• What issues can self-help help with? How should self-help services be structured?<br>• At what stage of the disease is self-help useful?<br>• What support could self-help offer in the end-of-life or bereavement phase?<br><br>• Are there certain aspects/circumstances/groups of people for whom self-help does not seem appropriate?<br>• Do you see any differences in the support provided to those affected themselves and to their relatives?
**Ideas for improving cooperation**	Would you like to see closer cooperation between self-help and hospice and palliative care?<br><br>What could facilitate cooperation/establishment of cooperation?	If yes:<br>• In what respect?<br>• At association or organizational level, or between institutions?<br>• Are there already concrete considerations in this regard within your association?<br>If no:<br>• Why not? Are there any obstacles?
**Outlook**	Is there anything else you would like to add that you think is important but has not been mentioned yet?	
**Sociodemographic data**	*Finally, we would like to ask you for a few details about yourself for statistical purposes:*<br>• Age	

	• Gender<br>• Highest level of education<br>• Occupation<br>• Family status<br>• Living situation (living alone, with family)<br>• Religion<br>• Nationality	
