# Supplementary material for: Interactions between self-help and hospice and palliative care – Opportunities, barriers and needs (Self-Pall): A study protocol
Source: PLoS One. 2026 Jul 9;21(7):e0350453. doi: 10.1371/journal.pone.0350453 (PMC13349143; doi:10.1371/journal.pone.0350453)
Supplement: S2 File — Care level. (PDF) [file pone.0350453.s002.pdf]

Self-Pall: Interview guide for representatives of Hospice and Palliative care (care level)

We are delighted to have you as an interview partner. My name is xx, and I will be conducting the interview with you today. The interview will take approximately 45 to 60 minutes.

We would like to talk about your experience working in hospice and palliative care with self-help groups (e.g., groups, organizations, associations). We have prepared a few questions that we would like to ask you.

Your experiences will help us to better understand the collaboration between hospice and palliative care and self-help groups. We want to use these findings in the Self-Pall project to develop recommendations for action. These recommendations are intended to strengthen and support cooperation between the two groups.

Do you have any questions before we start? We would like to record the interview so that we can transcribe it more accurately afterwards. Are you okay with that?

Consent forms filled out?

Turn on the recording device!

**Subject area**	**Questions**	**Check – was that mentioned? If not, ask for clarification**
**Description of the hospice and palliative care setup**	Could you start by briefly introducing the institution where you work and describing your role?	• Full-time or volunteer work?
**Self-disclosure**	*We would now be interested to know to what extent self-help plays a role in your work.*<br><br>First of all, what do you understand by self-help in the context of hospice and palliative care?	

**Experience with self-help in direct contact with patients**	*As we continue our conversation, we want to focus on health-related group self-help, where people with similar conditions come together and offer each other support*.<br><br>Have you cared for patients and relatives who have used such self-help services?	If yes:<br>• Can you please describe in more detail what you know about how this was for patients and their families?<br>• What kind of service was it?<br>• Do you know how the contact came about (family doctors)?<br>• When did it start in the course of the illness?<br>• In your opinion, what was helpful and what was less helpful or not helpful at all? Why?<br>• Was there also direct contact between your team and a representative of the self-help group?<br>If no:<br>• In your opinion, why do you think no patients/relatives took advantage of self-help services?<br>• Are you aware of any (other) suitable self-help services for your patients and their relatives?<br>If yes:<br>• Do you refer to these services? With what aim?
**Need for self-help in hospice and palliative care**	Do patients and relatives ask about self-help services?	• Do you see a need for self-help services in hospice and palliative care?<br>If yes:<br>• In what form? (e.g., information, contact mediation?)

**Possibilities and limitations of self-help for seriously ill people and their relatives**	*Now we would like to discuss the possibilities and limitations of self-help in more general terms.*<br><br>In your opinion, what kind of support could self-help provide in the area of hospice and palliative care?<br><br>What do you think self-help can and cannot achieve?<br><br>Do you know of any specific examples where self-help was helpful or hindering?	• What issues can self-help help with? How should self-help services be structured?<br>• At what stage of the illness is self-help useful?<br>• What support could self-help offer in the final stages of life or during the grieving process?<br><br>• Are there certain aspects/circumstances/groups of people for whom self-help does not seem appropriate?<br>• Do you see any differences in the support provided to those affected themselves and to their relatives?
**Ideas for improving cooperation**	Would you like to see closer cooperation between self-help and hospice and palliative care?<br><br>What could facilitate cooperation/achievement?	If yes:<br>• In what respect?<br>• At the association or organizational level, or between institutions?<br>• Are there already concrete considerations in your team or network?<br>If no:<br>• Why not? Are there any obstacles?
**Outlook**	Is there anything else you would like to add that you think is important but has not been mentioned yet?	
**Sociodemographic data**	*Finally, we would like to ask you for a few details about yourself for statistical purposes:*<br>• Age<br>• Gender<br>• Highest level of education<br>• Occupation<br>• Family status<br>• Living situation	

	• Religion<br>• Nationality	
