# Supplementary material for: Interactions between self-help and hospice and palliative care – Opportunities, barriers and needs (Self-Pall): A study protocol
Source: PLoS One. 2026 Jul 9;21(7):e0350453. doi: 10.1371/journal.pone.0350453 (PMC13349143; doi:10.1371/journal.pone.0350453)
Supplement: S3 File — (PDF) [file pone.0350453.s003.pdf]

Self-Pall: Interview guide for patients and relatives

We are delighted to have you as an interview partner. My name is xx, and I will be conducting the interview with you today. The interview will take approximately 45 to 60 minutes.

We would like to talk about your experience with self-help as someone affected by a serious illness or as a relative of someone who is seriously ill. We have prepared a few questions that we would like to ask you.

Your experiences will help us to better understand the collaboration between hospice and palliative care and self-help. We want to use these findings in the Self-Pall project to develop recommendations for action. These are intended to strengthen and support the collaboration between the two parties.

If you feel that a question is too personal and you do not wish to answer it, please let us know and we will skip that question. We understand that you are in a very sensitive situation and greatly appreciate that you are still taking the time to share your experiences with us.

Do you have any questions before we start? We would like to record the interview so that we can transcribe it more accurately afterwards. Are you okay with that?

Consent forms filled out?

Turn on the recording device!

**Subject area**	**Questions**	**Check – was that mentioned? If not, ask for clarification**
**Self-disclosure**	Could you start by briefly introducing your self-help group?	• Frequency of meetings, group size, online or in person<br>• How does a group meeting usually work?<br>• Can you briefly describe your condition?
	What motivates you to get involved in the self-help group?	• Was there a specific reason for joining the self-help group?

		• How long have you been in the group? At what stage of the disease did you join the self-help group?<br>• Did your relatives/the affected relative also contact self-help themselves?<br>• What support and assistance do you receive from the self-help group?<br>• Do you also take on specific tasks within the group?
	Additional information for group leaders:<br>What are your responsibilities as a group leader?	• How long have you been leading the group?<br>• Is your group structurally affiliated? (E.g., with an umbrella organization, other self-help groups, the city, hospital social services, family doctors)
	Now I would be interested to know what you mean by `self-help`?	*Note, in case of further inquiries: we are primarily interested in community-based (health-related) self-help, but if you have other experiences with hospice and palliative care, that is also of interest to us!*
**Dealing with dying, death, and grief in self-help**	*Introduction: Now we would like to talk about how your group deals with the topics of dying, death, and grief.*<br><br>Can you please describe how your group deals with the topics of dying, death, and grief?<br><br>Do you feel that the way the group deals with these topics is appropriate, or would you prefer something different?	• Is the topic generally discussed within the group, or is it avoided?<br>• How does the group deal with it when the health of individual group members deteriorates?<br>• Have any members of your group ever passed away? How was this handled?<br>• Are there any specific customs or rituals, for example?<br>• Specific examples?
	To group leaders:<br>Were you prepared for this?	• If so, how (e.g., training)?

<table>
<tr><td rowspan="3">Experience with hospice and palliative care</td><td>Now we would like to talk about hospice and palliative care.

Are hospice and palliative care services discussed as a form of support in your group?</td><td>• If so, how and what kind?
• In what form, e.g., written information, lecture, training?
• Are there any lectures? Is anyone from a relevant organization invited?
• Do you see a need for more information? Information provided by a group or family doctor?</td></tr>
<tr><td>Group leader:
What is your assessment: Is the term `hospice and palliative care` known within the group?</td><td></td></tr>
<tr><td>Have you had any personal experience with hospice and palliative care services?</td><td>• Are you aware of any hospice and palliative care services in your region (inpatient or at home)?
• How did you find out about them?
• If you have used these services: How/through whom did you make contact? Through your family doctor?
• If not: Who would you contact if you wanted information or were specifically looking for palliative care support?</td></tr>
<tr><td rowspan="2">Possibilities and limitations of self-help for seriously ill people and their relatives</td><td>Now we would like to discuss the possibilities and limitations of self-help groups for seriously ill people and their relatives.
Imagine that a disease is already at an advanced or even very advanced stage.

What support could your self-help group then provide for those affected? What can it not do?</td><td>• Can you give specific examples?
• What forms of support do you consider appropriate or inappropriate?</td></tr>
<tr><td>How could self-help support the relatives or grieving relatives of these people?</td><td>• Do you know of any specific examples where this has been successful?</td></tr>
</table>

		• Do you see any differences between support for those affected themselves and for their relatives?
**Cooperation with hospice and palliative care**	*Group leader:*<br>*Now we are interested in the interaction between self-help and hospice and palliative care. You lead the group and therefore probably also have insights into the association and organizational structures of your self-help association. We would now like to learn more about this.*<br><br>Is there any contact/cooperation between your self-help group and a hospice and palliative care institution?	• How did this come about?<br>• What is the goal of the collaboration?<br>• Where did the contact with the hospice and palliative care institution take place (function of contact person)?
Factors promoting and hindering cooperation	If yes: What works well and what doesn't work so well?<br><br>If no: Why not?	• Why is/was that the case?<br>• What would have been helpful?<br>• How could it have been done better?<br><br>• Was there a situation in which cooperation would have been helpful?<br>• What would be necessary for cooperation?
**Ideas for improving cooperation**	To everyone again:<br>When you think about your group and dealing with dying, death, and grief, what kind of support from hospice and palliative care would you find helpful?	• Are certain offers desirable for your self-help group?<br><br>Group leaders only:<br>Would support from professional organizations (hospice association) be helpful? Would closer cooperation be desirable? What could facilitate cooperation?

**Outlook**	Is there anything else you would like to add that you think is important but has not been mentioned yet?	
**Sociodemographic data**	Finally, we would like to ask you for a few details about yourself for statistical purposes:<br>• Age<br>• Gender<br>• Highest level of education<br>• Occupation<br>• Family status<br>• Living situation (living alone, with family)<br>• Religion<br>• Nationality	
