# Supplementary material for: Interactions between self-help and hospice and palliative care – Opportunities, barriers and needs (Self-Pall): A study protocol
Source: PLoS One. 2026 Jul 9;21(7):e0350453. doi: 10.1371/journal.pone.0350453 (PMC13349143; doi:10.1371/journal.pone.0350453)
Supplement: S4 File — (PDF) [file pone.0350453.s004.pdf]

Self-Pall: Interview guide for representatives of self-help groups (association level)

We are delighted to have you as our interview partner. My name is xx, and I will be conducting the interview with you today. The interview will take approximately 45 to 60 minutes.

In general, we are interested in the role that dying, death, and grief play in self-help and specifically in your work. We would also like to talk about any experience you may have gained in your work in the field of self-help with hospice and palliative care providers, e.g., hospices, outpatient palliative care services, or associations. We have prepared a few questions that we would like to ask you.

Your experiences will help us to better understand the cooperation between hospice and palliative care and self-help. We want to use these findings in the Self-Pall project to develop recommendations for action. These are intended to strengthen and support the cooperation between the two stakeholders.

Do you have any questions before we start? We would like to record the interview so that we can transcribe it more accurately afterwards. Are you okay with that?

Consent forms filled out?

Turn on the recording device!

Subject area	Questions	Check – was that mentioned? If not, ask for clarification
**Self-disclosure**	Could you start by briefly introducing your organization/association and explaining your role/responsibilities?<br>*Then, to begin with, I would be interested to know in general terms:*<br><br>What do you understand by \`self-help\`?	Full-time or volunteer?

	In the course of our discussion, we want to focus on health-related group self-help, where people affected by the same condition come together and offer each other support.	
**Dealing with dying, death, and grief in self-help**	Can you please describe how the topics of dying, death, and grief play a role in your work?	• Can you give specific examples?<br>• Do the self-help groups receive inquiries about dying/death/grief/end-of-life care?<br>• How does your organization deal with this issue?<br>• Are there any considerations at the association level to support your members in dealing with this issue (e.g., advising/informing/educating)?<br>• Do you see a need for this?
**Experience with hospice and palliative care**	Basically, we would like to learn more about the interaction between hospice and palliative care and self-help.<br><br>What do you understand by `hospice and palliative care`?	
	Does your organization/association discuss hospice and palliative care services as a form of support?<br><br>If so, how are they discussed and what specific services are offered?<br><br>If not, why not?	• What is being referred to, e.g., written information, website, lecture, training?<br>• Is someone from a relevant organization being invited?<br>• In your opinion, is there a need for this or not? Need in terms of what? Information material, knowledge transfer, training?<br>• How do you think hospice and palliative care is generally known within self-help?

**Possibilities and limitations of self-help** for seriously ill people and their relatives	*Now we are interested in what you think self-help can and cannot achieve in relation to advanced diseases and the end-of-life phase.*<br><br>In your opinion, what support could self-help offer people with advanced diseases and their relatives?<br><br>Can you give us some specific examples?	• What issues can self-help help with? How should self-help services be structured?<br>• At what stage of the disease is it useful?<br>• What support could self-help offer in the final stages of life or during the grieving process?<br>• Are there certain aspects/circumstances/groups of people for whom SH does not seem suitable?<br>• Do you see any differences in the support provided to those affected themselves and to their relatives?
**Cooperation between self-help and hospice and palliative care at association and organizational level**	*Now I would like to talk about possible cooperation at the organizational or association level:*<br><br>Is there already cooperation/contact with hospice and palliative care institutions at the association level?	If yes:<br>• What do the contacts look like? How did they come about (family doctors, inpatient hospice), when did they start? Is there a formal cooperation agreement?<br>• How would you describe the cooperation?<br><br>If no: Why? Are there any considerations in this regard?
**Factors promoting and hindering cooperation**	What works well, what doesn't work so well? Why?<br><br>*If there has been no cooperation so far:*<br><br>What would it take to establish/successful cooperation?	• Which framework conditions are helpful (city, municipality)?<br>• Which interfaces are relevant (social services in hospitals, family doctors, visiting services, hospital chaplaincies, care centers)?<br>• Is support from professional organizations (hospice associations) helpful?<br>• Structural level: how do the services actually interact?
**Ideas for improving cooperation**	When you think about your association/organization and how it deals with dying, death, and grief, what kind of support from hospice and palliative care would you find helpful?	• Would you like to see closer cooperation between self-help and hospice and palliative care?<br>• What could facilitate cooperation?

**Outlook**	Is there anything else you would like to add that you think is important but has not been mentioned yet?	
Sociodemographic data	Finally, we would like to ask you for a few details about yourself for statistical purposes:<br>• Age<br>• Gender<br>• Highest level of education<br>• Occupation<br>• Family status<br>• Living situation<br>• Religion<br>• Nationality	
