# Supplementary material for: Interactions between self-help and hospice and palliative care – Opportunities, barriers and needs (Self-Pall): A study protocol
Source: PLoS One. 2026 Jul 9;21(7):e0350453. doi: 10.1371/journal.pone.0350453 (PMC13349143; doi:10.1371/journal.pone.0350453)
Supplement: S5 File — (PDF) [file pone.0350453.s005.pdf]

Discussion guide

Objective: Coordination and revision of the first draft of recommendations for action regarding appropriate forms of self-help for patients and relatives in the context of hospice and palliative care, necessary personnel and structural requirements, and factors conducive to successful cooperation.

Target group/participants:

4-6 People plus moderation and transcript writer, participants from all target groups: 1) Patients and relatives with experience in self-help, 2) Representatives of hospice and palliative care (care and association level), 3) Representatives of self-help (association level)

Scheduled date: July 2026

Location: on site or online

Duration: 90-120 min.

Procedure

1. **Welcome** and introduction of the project team
2. Short sociodemographic questionnaire, participation list and consent forms
   - Data is only shared in encrypted form, i.e., pseudonymized—no traceability to participants
   - Audio recording for record
   - Questionnaire data to describe the participant group
3. Brief **project presentation** and **aim of the discussion**
   - The Self-Pall project investigates the interaction between hospice and palliative care and self-help and aims to strengthen cooperation between the two actors by developing recommendations for action
   - A first draft of recommendations for action was developed based on interview data.

   - The next step is to coordinate this initial draft of the recommended actions with you as representatives of patients and relatives, hospice and palliative care, and self-help, and to adjust it if necessary. That is the goal of today's discussion round.
4. Explanation of the established **communication rules**
5. **Introductions**
   - Please briefly introduce yourself (name, institution/ association, connection to the project)
6. Presentation of the **recommended actions developed by the project team**
   - We were able to derive xy recommendations for action from the interview data. We have selected the following recommendations for action in advance to discuss them with you today.
   - We have brought the following key questions as a basis for discussion:
7. **Key questions:**
   - To what extent can care be improved through networking and possible mutual referrals between hospice and palliative care and self-help?
   - What factors should play a key role?
   - What limitations should be taken into account?
   - At what structural level should measures be recommended?
8. **Summary of** the **discussion points** and the agreed **recommendations for action**
9. **Farewell**
   - Thank you very much for your willingness to participate in the discussion.
   - We will be happy to keep you informed about the further development of the recommendations for action and will send you a copy of the recommendations at the end of the project.
